# Insights into motor performance deficits after stroke: an automated and refined analysis of the lower-extremity motor coordination test (LEMOCOT)

**DOI:** 10.1186/s12984-021-00950-z

**Published:** 2021-10-26

**Authors:** Shirley Handelzalts, Yogev Koren, Noy Goldhamer, Adi Yeshurun-Tayer, Yisrael Parmet, Lior Shmuelof, Simona Bar-Haim

**Affiliations:** 1 Department of Physical Therapy, Recanati School for Community Health Professions, Faculty of Health Sciences, Ben-Gurion University of the Negev, P.O.B. 653, 84105 Beer-Sheva, Israel; 2 The Translational Neurorehabilitation Lab at Adi Negev Nahalat Eran, Ofakim, Israel; 3 Department of Industrial Engineering and Management, Ben-Gurion University of the Negev, Beer Sheva, Israel; 4 Department of Cognitive and Brain Sciences, Ben-Gurion University of the Negev, Beer Sheva, Israel

**Keywords:** CVA (cerebrovascular accident), Motor performance, Dexterity, Variable error, Absolute error, Neurological assessment

## Abstract

**Background:**

The lower-extremity motor coordination test (LEMOCOT) is a performance-based measure used to assess motor coordination deficits after stroke. We aimed to automatically quantify performance on the LEMOCOT and to extract additional performance parameters based on error analysis in persons with stroke (PwS) and healthy controls. We also aimed to explore whether these parameters provide additional information regarding motor control deficit that is not captured by the traditional LEMOCOT score. In addition, the associations between the LEMOCOT score, parameters of error and performance-based measures of lower-extremity impairment and gait were tested.

**Methods:**

Twenty PwS (age: 62 ± 11.8 years, time after stroke onset: 84 ± 83 days; lower extremity Fugl-Meyer: 30.2 ± 3.7) and 20 healthy controls (age: 42 ± 15.8 years) participated in this cross-sectional exploratory study. Participants were instructed to move their big toe as fast and accurately as possible between targets marked on an electronic mat equipped with force sensors (Zebris FDM-T, 60 Hz). We extracted the contact surface area of each touch, from which the endpoint location, the center of pressure (COP), and the distance between them were computed. In addition, the absolute and variable error were calculated.

**Results:**

PwS touched the targets with greater foot surface and demonstrated a greater distance between the endpoint location and the location of the COP. After controlling for the number of in-target touches, greater absolute and variable errors of the endpoint were observed in the paretic leg than in the non-paretic leg and the legs of controls. Also, the COP variable error differentiated between the paretic, non-paretic, and control legs and this parameter was independent of in-target counts. Negative correlations with moderate effect size were found between the Fugl Meyer assessment and the error parameters.

**Conclusions:**

PwS demonstrated lower performance in all outcome measures than did controls. Several parameters of error indicated differences between legs (paretic leg, non-paretic leg and controls) and were independent of in-target touch counts, suggesting they may reflect motor deficits that are not identified by the traditional LEMOCOT score.

**Supplementary Information:**

The online version contains supplementary material available at 10.1186/s12984-021-00950-z.

## Introduction

Motor coordination can be defined as the ability to produce context-dependent organized movements in spatial and temporal domains [1, 2]. During walking, relative motion between body segments needs to be adaptable to accommodate internal and external demands, in turn allowing for accurate foot placement and safe mobility [3–5]. Stroke survivors often demonstrate impaired motor coordination of the upper and lower extremities [6] that may cause limitations in the performance of daily activities, reduced participation, and decreased quality of life [7].

Performance-based measures of coordination for persons with neurological disorders are often based on time and criterion (e.g., Finger-to-Nose test [8], lower-extremity motor coordination test [9]), however, they do not quantify performance quality (i.e., how well movements are performed, whether they reflect a return toward premorbid pattern). In the Fugl Meyer Assessment, a commonly used measure to evaluate lower and upper extremity impairments after stroke, coordination is measured as the difference in time to alternately touch the targets five times between the more- and less-affected extremity [10]. In addition, the endpoint trajectory straightness/smoothness (tremor) and the precision (dysmetria) are estimated. However, the performance of these components is estimated on a 3-level rating scale [10], which limits the ability to detect and quantify small changes over time. A quantitative evaluation of the endpoint movement to target in terms of smoothness, straightness, error magnitude, speed and range of joint motion might provide a more refined and informative scale to characterize motor control deficits after stroke than merely time and criterion [11]. Here, we focus on quantifying the error (i.e., accuracy) of the performance in the lower-extremity motor coordination test (LEMOCOT) in persons with stroke (PwS).

The LEMOCOT is a performance-based measure of coordination [9]. In the test, performed while sitting, participants are instructed to move their lower extremity as fast and accurately as possible and alternately touch with their big toe a proximal and a distal target on the floor. The number of targets touched in 20 s constitutes the score. The LEMOCOT demonstrated appropriate measurement properties i.e. intra-, inter-rater, and test–retest reliability and construct validity in PwS [12]. In the current exploratory study, the LEMOCOT was performed on an electronic mat equipped with force sensors to quantify motor performance in terms of accuracy (i.e., endpoint absolute error) and consistency (i.e., endpoint variable error). Our assumption was that in a well-controlled movement, the endpoint location and the center of pressure (COP) location of the foot would be congruent to accurately reach the target, whereas in a less controlled movement (e.g., ‘throwing’ the leg towards the test’s targets) they would not. Therefore, we computed the accuracy and consistency for both—the endpoint location and the COP location. These measures might provide a more detailed and comprehensive assessment of motor deficits after stroke and may enable us to capture even subtle changes over time or in response to training interventions that may not be reflected in the traditional score (i.e., number of in target touches performed in 20 s). Furthermore, understanding how well PwS can perform targeted reaching with the paretic and non-paretic leg may be relevant for rehabilitation in terms of locomotor and balance control tasks, especially in activities where the margin for error in foot placement is small, such as negotiating cluttered travel paths or stepping over an obstacle.

For study purposes, we developed an algorithm to automatically compute the traditional LEMOCOT score of ‘in-target’ touch counts and calculate additional parameters of motor performance. Therefore, we aimed to (1) estimate the validity of our algorithm and script, (2) quantify motor performance in the LEMOCOT using parameters of error in PwS and healthy controls, (3) investigate whether these parameters provide different or additional information to that provided by the traditional score, and finally, (4) to determine the association between the traditional LEMOCOT score, parameters of error and performance-based measures of lower extremity motor impairments and gait.

## Methods

### Participants

Twenty PwS and 20 healthy controls participated in this cross-sectional study (Table 1). PwS were recruited during their hospitalization at the Adi Negev Nahalat-Eran Rehabilitation Center in Ofakim, Israel. Inclusion criteria for PwS included first-ever unilateral ischemic or hemorrhagic stroke and being able to voluntarily extend and bend the affected leg to reach the test’s targets. Exclusion criteria included other musculoskeletal or neurological injuries, pain that could interfere with the performance of the tasks, and clinical instability. The control group was recruited among staff and had no known musculoskeletal or neurological movement disorders. All participants signed an informed-consent form. The study was approved by the Regional Ethical Review Board at Sheba Medical Center, Israel (Approval Number 6218-19-SMC).

**Table 1:** Participants’ characteristics

Characteristic	Stroke (n = 20)	Control (n = 20)
Age, years	65.0 (41–83)	35.5 (26–73)*
Sex (male/female), n	3/17	13/7*
Height, cm	169.1 ± 9.9	166.3 ± 9.7
Weight, kg	77.4 ± 17.4	69.5 ± 15.4
Time after onset, days	49.0 (9–279)	N/A
Stroke type (Ischemic/Hemorrhagic), n	14/6	N/A
Stroke side (Right/Left), n	14/6	N/A
Fugl-Meyer lower extremity (0–34)	31.5 (20–34)	N/A
Timed up & go test, sec	15.3 (7.15–43.7)	N/A
10 m walk test, m/s	10.4 (7.8–34.7)	N/A

Values are mean ± SD for continues variables with normal distribution and median (range) for ordinal or non-normally distributed variables. *p < 0.05 for between group comparison

### Procedure

**Fig. 1:**
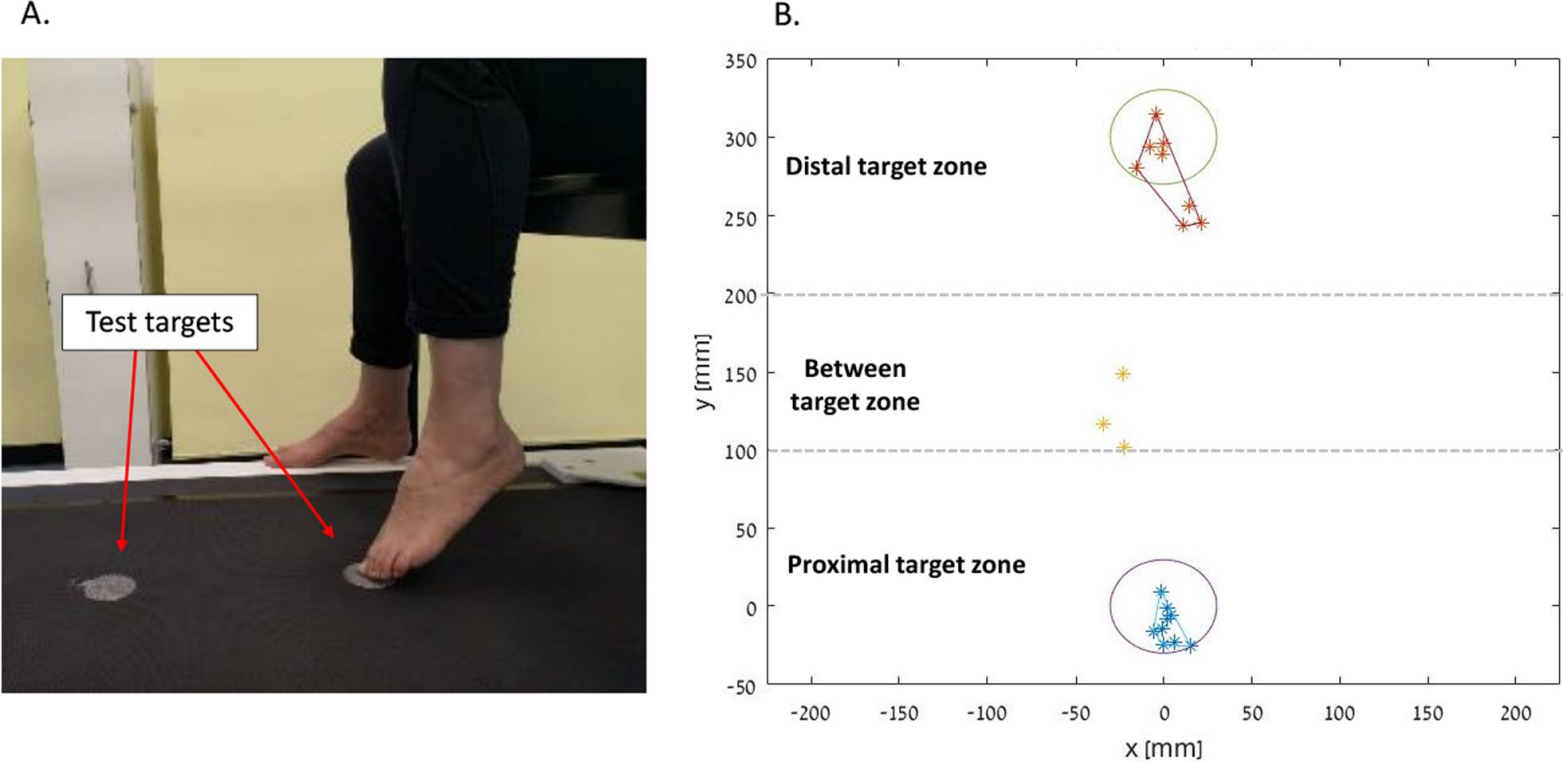
Study setup (**A**); The algorithm divides the tested surface into 3 zones: *the proximal target zone* (100 mm from the center of the proximal target (i.e., the origin) on the y axis); *the distal target zone* (100 mm from the center of the distal target on the y axis) and the *between target zone*. The blue and red dots represent touches included in the analysis of the proximal and distal target respectively. The yellow dots represent touches that were between the targets. For example, in the proximal target all 9 touches were identified as ‘in-target’ whereas in the distal target 5 touches were considered as ‘in-target’ and 3 were considered as ‘outside-target’. Three touches were identified as ‘between-targets’ (**B**)

The proximal and distal targets were marked 30 cm apart [9] on an electronic mat with 10,240 miniature force sensors, each 0.80 × 0.80 cm (Zebris FDM-T Treadmill, *Zebris Medical GmbH*, Germany). Force data was acquired at 60 Hz sampling frequency using the software provided by the manufacturer (Zebris FDM, version 1.18.40). Before testing, the origin of axes was set at the center of the proximal target. Participants sat on a chair with a seat height of 44 cm and preformed the test barefoot, as described by Desrosiers et al. [9]. After a familiarization trial, participants were instructed to alternately touch the proximal and distal targets with their big toe as fast and as accurately as possible for 20 s. PwS performed the test first with their non-paretic leg, followed by their paretic leg [9] and healthy participants performed the test first with their dominant leg (i.e., the leg used to kick a ball), followed by their non-dominant leg (Fig. 1A). During the test, a physical therapist counted the number of in-target touches.

### Data processing

A dedicated algorithm and a MATLAB script (MathWorks Inc.) were developed and used to analyze force data. A detailed description of the algorithm can be found in the Additional file 1. Briefly, the script determines the location of the big toe (i.e., the endpoint) from the distribution of the force data. To determine the time of touch, we used the first time point at which the endpoint location was closest to target’s center. Since in many cases a participant’s foot touched the surface on the way toward the target (i.e., between targets), we divided the tested area into three zones: the ‘proximal target zone’, the ‘distal target zone’, and the ‘between target zone’ (Fig. 1B). For touches detected in the proximal and distal zones we determined whether the endpoint fell within or outside the limits of the target. A touch detected in the ‘between target zone’ was considered a ‘between’ target touch. Then, the total ‘in’, ‘out’, and ‘between’ target touch counts were calculated.

For each of the ‘in’ and ‘out’ touches we computed (1) the *touch’s surface* (i.e., the surface contacting the ground at the time of touch); (2) the coordinates of the endpoint position and the center of pressure (COP) position at the time of touch (i.e., the first time point at which the endpoint location was closest to target’s center) (3) the Euclidian distance between these coordinates (i.e., the distance between endpoint and COP location, Additional file 1: Fig. S1); (4) the absolute error which was calculated separately for the endpoint and COP position in relation to target’s center; and 5) the *variable error*— as the distance from the mean position of all repetitions in the same leg and target computed for each endpoint and COP position.

$${\text{Absolute error}}:\sqrt {\left(X_{i } - X_{target} \right)^{ 2} + \left( {Y_{i} - Y_{target} } \right)^{ 2} }$$

$${\text{Variable error}}:\sqrt {\left(X_{i } - \overline{X}\right)^{ 2} + \left( {Y_{i} - \overline{Y}} \right)^{ 2} }$$

### Statistical analysis

Statistical analysis was performed using SPSS (version 26.0). To estimate the validity of our algorithm and script, we first tested the correlation between the tester’s count and the count produced by our script. For a perfect agreement, a linear correlation with a slope of one and a constant term equal to zero is expected. Since for each participant we had two measurements (one for each leg) we used a mixed-effect model with the *tester’s count* as the dependent variable, *script count* as the fixed effect, and *subject* as the random effect. To estimate the strength of the correlation we calculated the conditional pseudo R^2^ [13]. In addition, we estimated the agreement between methods.

To test the effects of group and leg on the performance in the LEMOCOT, we used mixed-effect models with ‘participants’ as the *random* effect. In all cases, full factorial models with ‘group’ (stroke/controls) and ‘leg’ (paretic/non-paretic/dominant/non-dominant) as main effects were used. In several cases, the dependent variable and the ‘in-target’ touch counts correlated. Therefore, we controlled for the ‘in-target’ touch count by using the data from healthy controls to create a prediction model. Specifically, when we tested the correlation between these parameters within the control group, we found a positive relation, consistent with the speed-accuracy tradeoff concept [14]. Since PwS generally performed more slowly (i.e. fewer touches in 20 s), their error was expected to decrease in comparison with controls. Thus, we controlled for the number of touches by calculating the difference between the observed and the model-based predicted values. These ‘residuals’ (of all participants) were then tested as the dependent variable. Significance level was set at α < 0.05 and the sequential Bonferroni method was used to correct for multiple comparisons when appropriate. The models’ residuals were evaluated for their distribution. For all parameters, a logarithmic transformation [denoted as Ln(Parameter)] was used to overcome a violation of the requirement of normal distribution.

The associations between the LEMOCOT score (in-target touches), error parameters of the paretic leg and performance-based measures of motor impairments and mobility (i.e., Fugl Meyer Assessment, Timed Up & Go test and 10 m walk test) were tested using the Spearman’s *ρ* correlation coefficients, with *r* of 0.10 interpreted as a small effect, 0.30 as a medium effect, and 0.50 as a large effect [15].

## Results

### Algorithm and script validation

Significant correlation between the examiner’s count and the count produced by the script was found (p < 0.001) with a slope that was not significantly different from one ($\hat{\beta }$ = 1.03, 95% CI 0.96–1.10) but with a constant term that was significantly different from zero ($\hat{\beta }$ = 1.76, 95% CI 0.01–3.50), indicating a bias. Calculating the conditional pseudo R^2^ revealed that this model explained 97% of the variance in the sample, indicating that the algorithm produced valid results. Agreement between methods demonstrated a median difference of 1.5 touches with limits of agreement (i.e., 95% CI of the measurement) between −1 and 10.9. Also, we found that the difference between methods increased as a function of the examiner’s count ($\hat{\beta }$ = 0.1, p = 0.001).

### Parameters of motor performance

**Fig. 2:**
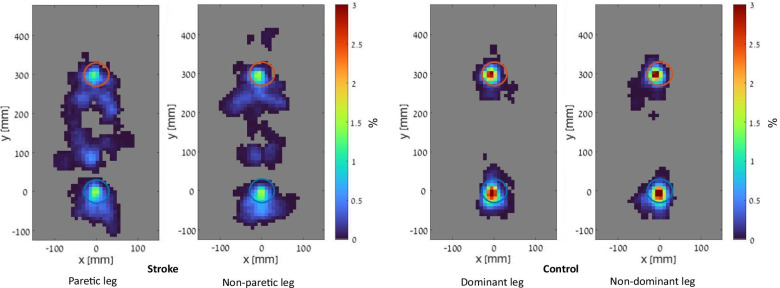
Density plots of the surface distribution in each touch, by group and leg (resolution of 8.4 mm^2^). Units: Percentage of touches in the colored region, with warmer colors indicating greater number of touches. The blue and red circles represent the proximal and distal target respectively

The number of ‘in-’, ‘out-’ and ‘between-’ target touches by leg are reported in Table 2. Density plots of the contact surface distribution by leg are presented in Fig. 2. One participant in the stroke group performed only one touch in each target with the paretic leg. Therefore, the variable error of the paretic leg could not be computed for this participant.

**Table 2:** Frequency of in-, out- and between- target touches (n) by leg

Group	Leg	In	Out	Between
Stroke	Paretic	13.0 ± 6.2 12.5 (1–25)	2.2 ± 2.3 1.5 (0–7)	4.4 ± 3.8 5.0 (0–16)
	Non-paretic	17.2 ± 7.2 15.5 (6–31)	1.7 ± 2.3 1.0 (0–7)	3.2 ± 3.9 1.5 (0–11)
Controls	Dominant	34.7 ± 10.9 35.0 (11–53)	1.0 ± 1.5 0.5 (0–5)	0.5 ± 1.0 0.0 (0–3)
	Non-dominant	33.1 ± 11.0 30.0 (13–55)	1.2 ± 2.0 0.5 (0–8)	0.2 ± 0.7 0.0 (0–3)

Results are reported as mean ± SD and median (range)

### In target touches (the traditional score)

**Fig. 3:**
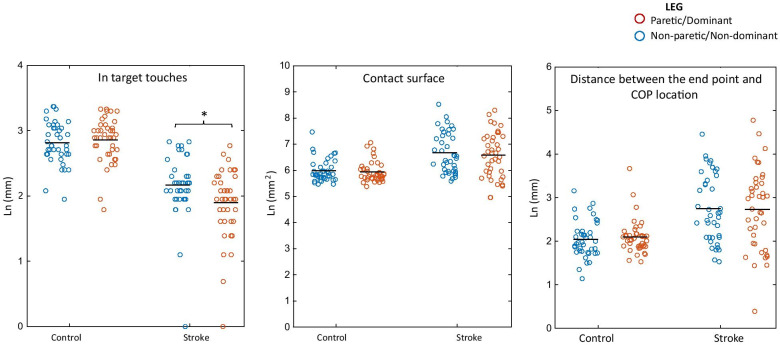
Motor performance parameters by group and leg. Significant main effect for group was found for all parameters indicating lower performance in persons with stroke than in controls. Significant group by leg interaction was found for in-target touches (*p < 0.001)

Significant main effects for group (F_1,156_ = 47.8, p < 0.001) and leg (F_1,156_ = 6.5, p = 0.012) and a significant group by leg interaction (F_1,156_ = 12.4, p = 0.001) were found. Results indicated that controls performed more ‘in-target’ repetitions than PwS (2.8 ± 0.08SE vs. 2.0 ± 0.08SE) and that participants performed more repetitions with their non-paretic/non-dominant leg than with the paretic/dominant leg (2.5 ± 0.06SE vs. 2.4 ± 0.06SE). Post hoc pairwise comparisons revealed a mean difference of 0.26 ± 0.06SE (p = 2.97 × 10^–5^) between the non-paretic and the paretic leg in PwS (2.2 ± 0.09SE vs 1.9 ± 0.09SE) while no significant difference was found between the dominant and non-dominant leg in controls (2.8 ± 0.09SE vs. 2.8 ± 0.09SE, p = 0.493), indicating that the main effect for ‘leg’ was derived exclusively from the performance of PwS (Fig. 3).

### Contact surface

A significant main effect for group (F_1,156_ = 14.5, p < 0.001) was found, indicating a greater contact surface in PwS than in controls (6.6 ± 0.12SE vs 5.9 ± 0.12SE). No significant main effect for leg (F_1,156_ = 1.1, p = 0.290) or group by leg interaction (F_1,156_ = 0.12, p = 0.727) were found (Fig. 3).

### The distance between the endpoint and the COP location

A significant main effect for group (F_1,156_ = 15.3, p < 0.001) was found, indicating a greater distance between the endpoint and the COP location in PwS than in controls (2.74 ± 0.1SE vs. 2.07 ± 0.1SE). No significant main effect for leg (F_1,156_ = 0.057, p = 0.812) or significant effect for the group by leg interaction (F_1,156_ = 0.218, p = 0.642) were found (Fig. 3).

### Endpoint absolute error

**Fig. 4:**
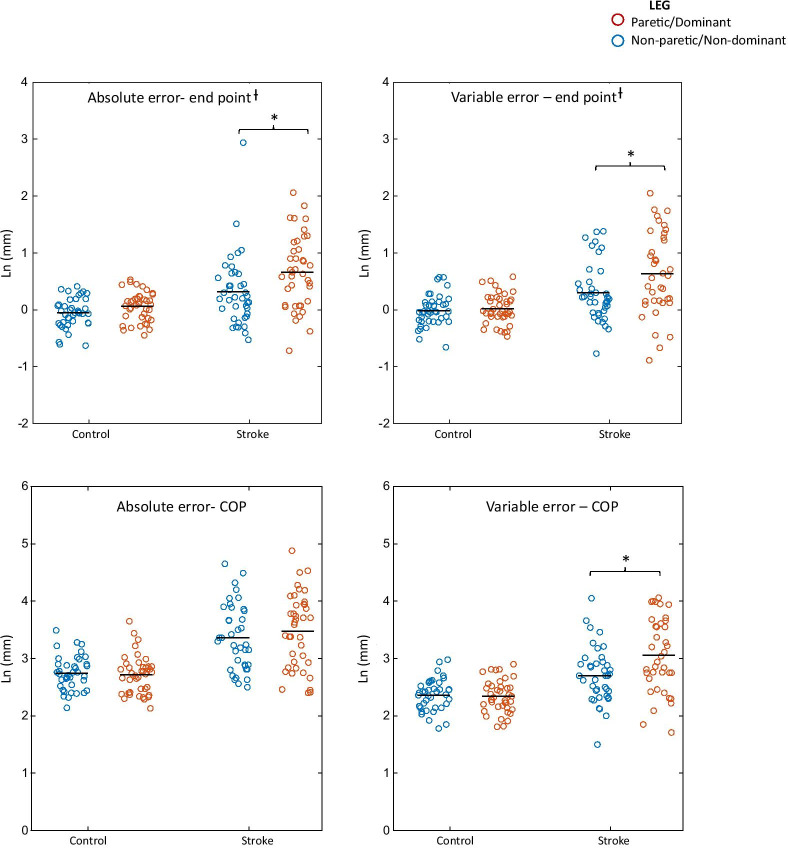
Performance error and variability parameters by group and leg. Significant main effect for group was found for all parameters indicating increased error and variability in persons with stroke (PwS) than in controls. Significant group by leg interaction was found for the end point variable error and the COP variable error indicating greater variability in the paretic leg than in controls. *post-hoc comparisons demonstrating significant differences between the paretic and non-paretic leg in PwS and between the non-paretic leg and control legs (p < 0.05); ^Ɨ^ Values after controlling for number of in-target touches. Abbreviations: COP, center of pressure

Significant main effects for group (F_1,156_ = 7.1, p = 0.008) and leg (F_1,156_ = 11.6, p = 0.001) were found. Results indicated that the mean absolute error was greater in PwS than in controls (2.72 ± 0.06SE vs. 2.48 ± 0.06SE) and greater in the paretic/dominant leg than in the non-paretic/non-dominant leg (2.7 ± 0.05SE vs. 2.5 ± 0.06SE). No significant group by leg interaction was found (F_1,156_ = 1.4, p = 0.233). However, this parameter correlated with the number of in target touches. After controlling for the number of in target touches, as was described in the methods, we found significant main effects for group (F_1,156_ = 23.7, p < 0.001) and leg (F_1,156_ = 12.0, p = 0.001). Although the interaction term in the controlled model was still non-significant (F_1,156_ = 3.05, p = 0.082), post hoc pairwise comparisons revealed significant differences between the paretic and non-paretic leg in PwS (p < 0.001) and between the non-paretic leg in PwS and the non-dominant leg in controls (p = 0.002) while no significant difference between legs was found in controls (p = 0.226) (Fig. 4).

### Endpoint variable error

Significant main effects for group (F_1,154_ = 4.8, p = 0.030) and leg (F_1,154_ = 8.1, p = 0.005) and a significant group by leg interaction (F_1,154_ = 4.2, p = 0.042) were found. Results indicated that the mean variable error was greater in PwS than in controls (2.4 ± 0.08SE vs. 2.1 ± 0.08SE) and greater in the paretic/dominant leg than in the non-paretic/non-dominant leg (2.3 ± 0.06SE vs. 2.2 ± 0.06SE). Post hoc pairwise comparisons revealed a mean difference of 0.36 ± 0.12SE (p = 0.005) between the paretic leg and control legs (2.5 ± 0.09SE vs. 2.2 ± 0.09SE) while no significant difference was found between the non-paretic leg and the non-dominant leg (2.2 ± 0.09SE in both legs of controls, p = 0.276). Nevertheless, this parameter also correlated with the number of in target touches. After controlling for the number of in target touches, we found significant differences between the paretic leg and the non-paretic leg (p < 0.001) and between the non-paretic leg and the non-dominant leg (p = 0.014) while no significant difference was found between legs in controls (p = 0.703) (Fig. 4).

### COP absolute error

Significant main effect for group (F_1,156_ = 30.7, p < 0.001) was found while the main effect for leg (F_1,156_ = 0.67, p = 0.414) and the group by leg interaction (F_1,156_ = 1.96, p = 0.163) were non-significant. Results indicated that the mean absolute error was greater in the PwS compared with controls (3.4 ± 0.09SE vs. 2.7 ± 0.09SE) (Fig. 4).

### COP variable error

Significant main effect for group (F_1,154_ = 23.1, p < 0.001), leg (F_1,154_ = 9.0, p = 0.003), and group by leg interaction (F_1,154_ = 11.8, p = 0.001) were found. Results indicated that the mean variable error was greater in the PwS than in controls (2.87 ± 0.07SE vs. 2.35 ± 0.07SE) and greater in the paretic/dominant leg compared with the non-paretic/non-dominant leg (2.69 ± 0.06SE vs. 2.53 ± 0.06SE). Post hoc pairwise comparison revealed a mean difference of 0.34 ± 0.07SE (p = 1.3 × 10^–5^) between the paretic and non-paretic leg (3.05 ± 0.08SE vs. 2.70 ± 0.08SE) while no significant differences were found between the dominant and non-dominant leg in controls (p = 0.756) (Fig. 4).

### Associations between the LEMOCOT score, paretic leg’s error and performance-based measures of motor impairments and gait

Negative correlations with moderate effect size were found between the Fugl Meyer assessment and the endpoint variable error (r = −0.348, p = 0.037), COP absolute error (r = −0.384, p = 0.021) and COP variable error (r = −0.427, p = 0.009). Negative correlations with large effect size were found between the traditional LEMOCOT score (i.e., in target touches) and the Timed Up & Go (r = −0.617, p < 0.001) and 10 m walk tests (r = −0.496, p = 0.002).

## Discussion

In this study we aimed to quantify motor performance deficits in the LEMOCOT using parameters of performance error in persons with stroke (PwS). We found that PwS performed worse than controls in all parameters: they touched the targets with greater foot surface, demonstrated a greater distance between the endpoint location and the location of the COP, and showed difficulties in placing both feet accurately and consistently on test targets. These motor performance parameters were found to add information beyond that provided by the traditional score of ‘in-target’ touch counts, as these parameters either did not relate to the traditional score or differed between groups and legs after controlling for the number of in-target touches.

Our first aim was to automatically compute the traditional LEMOCOT score of in-target touches. We found a significant correlation between the examiner’s count and the counts identified by our algorithm. This correlation explained 97% of the variance in the sample, indicating that our algorithm produced valid results. This is of great importance as test results can be computed without the dependency on the tester. However, an estimation of the agreement between methods, along with the positive constant term in the linear model, revealed that the tester’s count was consistently greater than the count produced by our software. Furthermore, the difference between methods increased as a function of the count. These findings might point to examiner’s difficulty in counting in-target touches accurately rather than the total movements (i.e., in- and out- target touches) performed in the LEMOCOT. This difficulty is likely to be more prominent as movements become faster, increasing the examiner’s cognitive load and causing a speed-difference relation, as was observed. Although this difficulty is unlikely to be prominent in populations with neurological disorders as they perform slowly, the automated algorithm may mitigate this issue in other populations.

Our second aim was to quantify parameters of error in the lower-extremity motor coordination test (LEMOCOT). Our exploratory analyses included several parameters that are considered to reflect the accuracy and variability of the motor output. We hypothesized that under normal, healthy conditions the foot’s surface contacting the ground would be small (representing exclusively the surface of the big toe) and that the endpoint location and the COP location would be congruent. These assumptions are reflected in the instructions given to the participants (i.e., touch the targets with the big toe as fast and as accurately as possible). Indeed, our results support these assumptions as we found that the mean contact surface and the mean distance between the endpoint location and the COP location were greater in PwS than in controls. Findings suggest a more gross control/less dexterous control or a compensatory strategy that was used to place the big toe on the target. Also, other parameters used in this study which are commonly used measures to quantify error were found to differ between groups and legs. Specifically, we found significant differences between the paretic leg, non-paretic leg and controls for the endpoint absolute error, endpoint variable error and COP variable error. For the COP absolute error, we noticed a smaller error for the non-paretic leg in comparison to the paretic leg, however, it did not reach significance level. This may be a result of statistical power (i.e., small sample size) or that this parameter reflects a deficit that is not side specific. Thus, this lack of effect needs to be examined in future studies.

In addition, we observed that PwS touched the surface on the way towards the target (i.e., between target zone, Table 2), behavior that might reflect another unexplored aspect of lower limb coordination within the context of the LEMOCOT. Studies exploring the touch behavior of people with motor impairments while interacting with a touch screen have shown that multiple fingers and various parts of the hand touched the screen [16]. Trewin et al. [17], reported that users with motor impairment slide their finger along the screen for stability as they approach the target. Our observations, along with these previous reports, suggest that lower-extremity ‘touch behavior’ is possibly important and should be further explored in future studies.

In line with previous results, significant lower scores in the LEMOCOT were observed in the non-paretic leg than in the controls [18]. In the current study, this reduction was accompanied by greater magnitude of error. Moreover, this increase in the endpoint absolute error and variable error was at least partially independent of the count score, indicating that these measures provide information that is not reflected by the traditional score. Deficits in motor coordination and dexterity of the non-paretic upper-extremity [19–21] and lower extremity [19] were previously observed in PwS. It was suggested that the ipsilesional extremity is not exclusively controlled by the contralesional hemisphere [22]; therefore, unilateral brain damage is expected to affect both extremities, as was observed. Interestingly, it has been shown that the kind of ipsilateral deficit seen depends on the side of stroke: left hemisphere damage was associated with directional trajectory errors while right hemisphere damage was associated with endpoint errors [22]. In our cohort, 70% of PwS had a lesion in the right hemisphere, which might contribute to the increased endpoint absolute and variable error observed in our results. Nevertheless, exploring the difference between hemispheres is beyond the scope of this study and should be further explored in the future.

While most of our parameters differentiated between groups (stroke vs. controls), they were also independent of the traditional LEMOCOT score. This points to their potential to add information regarding changes in motor coordination/control that are not captured by the traditional LEMOCOT score. Although none of these parameters was more sensitive to deficit than was the traditional count score (differentiating between groups and legs), we believe they have the potential to detect more subtle changes than the count as the latter quantifies in an ‘all-or-nothing’ fashion. Future studies should examine the sensitivity of these measures to the dynamics of motor recovery after stroke as reflected by changes in daily life functions such as gait.

Our fourth aim was to test the association between the LEMOCOT score, parameters of error of the paretic leg and performance-based measures of motor impairments and gait. Interestingly, measures of gait (i.e., TUG and 10 m walk test) demonstrated large negative associations with the traditional LEMOCOT score of in-target touches, but not with the parameters of error. On the other hand, the Fugl Meyer assessment of the lower-extremity demonstrated moderate, negative association with parameters related to performance error (endpoint variable error, COP absolute error and COP variable error), but not with the traditional LEMOCOT score. The Fugl Meyer is a measure of motor control and strength which may support our hypothesis that our measures provide additional data regarding motor control deficits. On the other hand, measures that assess the time required to walk a certain distance do not directly assess motor impairments which may explain why we did not find correlations with measures of performance error. In future studies it might be interesting to examine the associations between our parameters and parameters that are thought to reflect motor control of walking such as spatiotemporal variability.

The study has several limitations. First, our control group was not age matched to the PwS group. In a previous study among PwS (n = 106) [23], motor recovery of the lower extremity (i.e., assessed by the Fugl Meyer assessment) and age were significant predictors of the LEMOCOT score in the paretic leg. However, motor recovery of the lower extremity alone explained 46% of the variance in the LEMOCOT score while age added only 3% of the explained variance. Nevertheless, due to this age difference we cannot conclude with certainty that the difference between groups was derived exclusively from deficits caused by brain damage. Second, a touch represents an entire area, and therefore we couldn’t know where the participant was intending to touch. It was our assumption that the intended touch location is the one when the force was the closest to target’s center, an assumption that is not necessarily true and may have caused us to over-estimate the participants’ true abilities.

## Conclusions

Findings indicate that PwS preformed worse than controls in all outcome measures: they touched the targets with greater foot surface, demonstrated a greater distance between the endpoint location and the location of the COP, had greater errors and their performance was more variable. Several parameters of error detected differences between legs (paretic leg, non-paretic leg and controls) and were independent of in-target touch counts, suggesting they may reflect motor deficits that are not identified by the traditional LEMOCOT score.

## Supplementary Information

**Additional file 1: Fig. S1.** The Euclidian distance between the endpoint (red dot) and COP (black dot) coordinates in each touch was calculated to compute the distance between the endpoint and COP locations.

## Data Availability

The datasets used and/or analyzed during the current study are available from the corresponding author on request.

## Abbreviations

COP: Center of pressure.
LEMOCOT: Lower extremity motor coordination test.
PwS: Persons with stroke
