# Supplementary material for: Insights into motor performance deficits after stroke: an automated and refined analysis of the lower-extremity motor coordination test (LEMOCOT)
Source: J Neuroeng Rehabil. 2021 Oct 26;18:155. doi: 10.1186/s12984-021-00950-z (PMC8549232; doi:10.1186/s12984-021-00950-z)
Supplement: Supplementary file 1 — Additional file 1: Fig. S1. The Euclidian distance between the endpoint (red dot) and COP (black dot) coordinates in each touch was calculated to compute the distance between the endpoint and COP locations. [file 12984_2021_950_MOESM1_ESM.docx]

**Additional file 1**

A dedicated algorithm and a software were developed for study purposes. First, the x and y axes of the raw data were calibrated according to the device origin located at the center of the proximal target. Since in many cases a participant’s foot touched the surface on the way toward the target, we divided the tested area into three zones: the ‘proximal target zone’ [100mm from the center of the proximal target (i.e., the origin) on the y axis], the ‘distal target zone’ (100 mm from the center of the distal target on the y axis), and the ‘between target zone’ (Figure 1 in the manuscript).

For force that was detected in the proximal or distal target zone, we used the force’s most distal and medial location to estimate the location of the big toe (i.e., the endpoint). To determine the time of touch, we used the first time segment where the endpoint was the closest to target’s center (i.e., the distance between the two points). For touches detected in the proximal and distal zones we determined whether the endpoint fell within or outside the limits of the target. A touch detected in the ‘between target zone’ was considered a ‘between’ target touch. Then, the total ‘in-’, ‘out-’ and ‘between-’ touch counts were calculated.

For each ‘in’ and ‘out’ touches we computed: 1) the *touch’s surface* (i.e., the surface contacting the ground at the time of touch); 2) the coordinates of the endpoint position and the center of pressure (COP) position:

$$\mathrm{COP}ֹ_{X}=\frac{\sum_{i,j} x_{i}F_{i,j}}{\sum_{i,j} F_{i,j}} , \mathrm{COP}ֹ_{Y}=\frac{\sum_{i,j} y_{i}F_{i,j}}{\sum_{i,j} F_{i,j}}$$

3) the Euclidian distance between these coordinates (i.e., the distance between endpoint and COP location) (Figure 1, supplementary).

4) the absolute error which was calculated separately for the endpoint and COP position in relation to target’s center:

Absolute error: $\sqrt{{{(X}_{i}-X_{target})}^{2}+{(Y_{i}-Y_{target})}^{2}}$

5) the *variable error*— as the distance from the mean position of all repetitions in the same leg and target— for each endpoint and COP position:

Variable error: $\sqrt{{{(X}_{i}-\bar{X})}^{2}+{(Y_{i}-\bar{Y})}^{2}}$


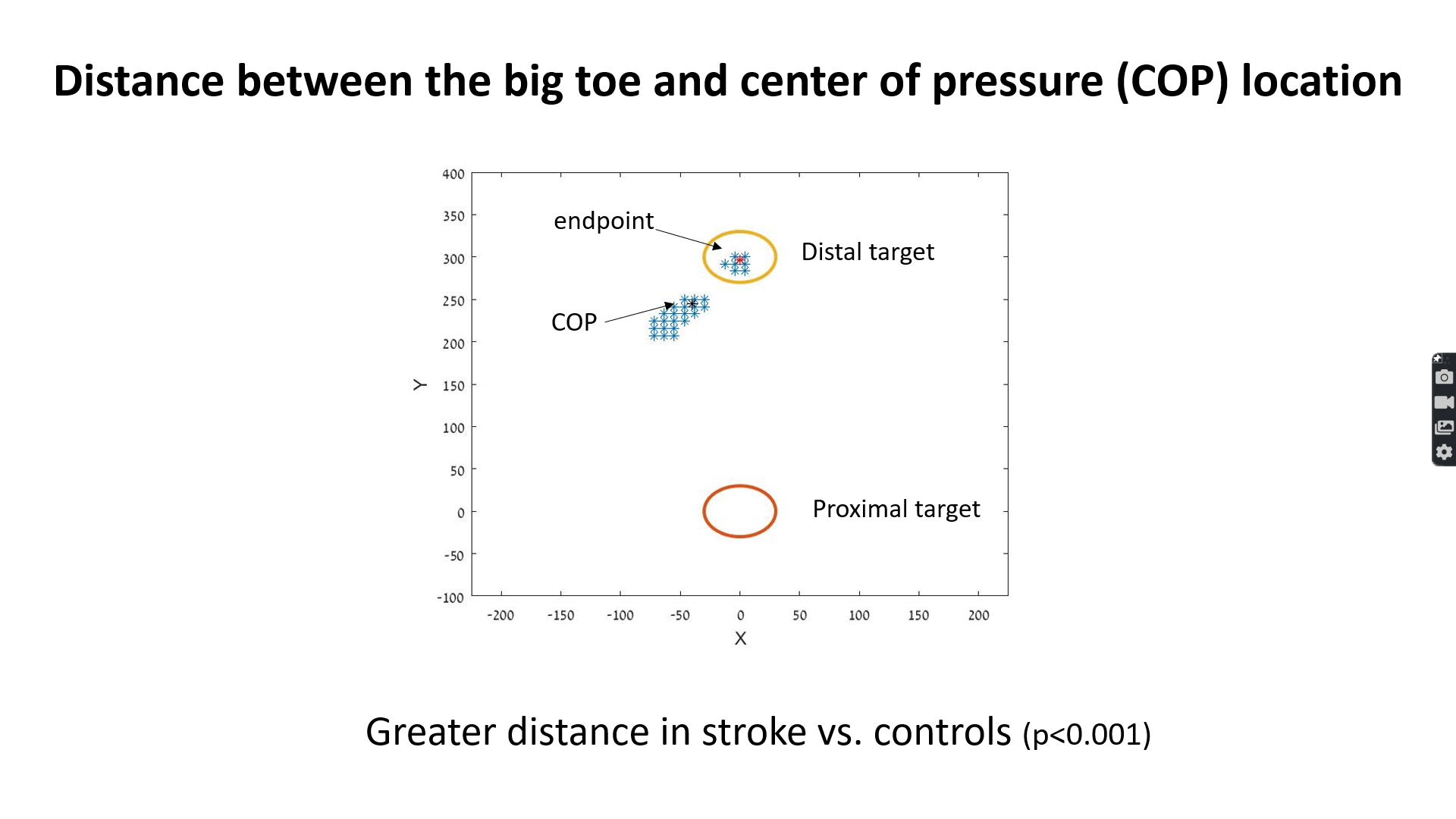


**Figure S1.** The Euclidian distance between the endpoint (red dot) and COP (black dot) coordinates in each touch was calculated to compute the distance between the endpoint and COP locations.
